# Long-term outcome of patients with long-standing persistent atrial fibrillation undergoing ablation guided by a novel high-density panoramic mapping system: A propensity score matching study

**DOI:** 10.1016/j.hroo.2022.04.003

**Published:** 2022-04-19

**Authors:** Ting-Yung Chang, Chin-Yu Lin, Yenn-Jiang Lin, Cheng-I Wu, Shih-Lin Chang, Li-Wei Lo, Yu-Feng Hu, Fa-Po Chung, Ta-Chuan Tuan, Tze-Fan Chao, Jo-Nan Liao, Ling Kuo, Chih-Min Liu, Shih-Ann Chen

**Affiliations:** ∗ Heart Rhythm Center, Division of Cardiology, Department of Medicine, Taipei Veterans General Hospital, Taipei, Taiwan; † Division of Cardiology, Taipei Veterans General Hospital, Taipei, Taiwan; ‡ Institute of Clinical Medicine, and Cardiovascular Research Institute, National Yang Ming Chiao Tung University, Taipei, Taiwan; § National Taipei University of Nursing and Health Sciences, Taipei, Taiwan; ¶ Cardiovascular Center, Taichung Veterans General Hospital, Taichung, Taiwan

**Keywords:** Ablation, CARTOFINDER, Long-standing persistent atrial fibrillation, Outcome, Substrate modification

## Abstract

**Background:**

Catheter ablation is a current therapeutic approach for atrial fibrillation (AF). However, its efficacy for long-standing persistent AF remains suboptimal.

**Objective:**

The purpose of this study was to test the hypothesis that a panoramic mapping system (CARTOFINDER, Biosense Webster) can guide pulmonary vein (PV) isolation and additional potential AF drivers.

**Methods:**

A total of 76 patients with nonparoxysmal AF referred for ablation guided by a novel high-density panoramic mapping system with CARTOFINDER were prospectively enrolled. Of this cohort, 40 patients (52.6%) had long-standing persistent AF (CARTOFINDER group). We then retrospectively screened the patients with long-standing persistent AF undergoing conventional PV isolation and elimination of non-PV triggers during the contemporary period (conventional group). They were matched at a 1:2 ratio (40 patients in group 1 received ablation guided by CARTOFINDER; 80 patients in group 2 receiving conventional PV isolation and elimination of non-PV triggers).

**Results:**

During follow-up, patients in group 1 had a lower recurrence AF rate than those in group 2 (*P* = .040). There was no difference in recurrence of atrial flutter (*P* = .996) and atrial tachycardia (*P* = .525). In Cox proportional hazards regression analysis, AF duration and PV isolation along with AF driver ablation using a panoramic mapping system with CARTOFINDER both were independent predictors of recurrent AF after catheter ablation of long-standing persistent AF.

**Conclusion:**

Identification of the potential drivers in long-standing AF is crucial. Compared with conventional PV isolation and elimination of non-PV triggers, ablation guided by a high-density panoramic mapping system (CARTOFINDER) might have a better outcome in patients with long-standing persistent AF.

**Key Findings**

▪ Mapping with CARTOFINDER can identify focal and rotational activities and assist in modifying wide-area pulmonary vein (PV) ablation.
▪ The stepwise approach of the CARTOFINDER method resulted in less recurrent atrial fibrillation (AF) in patients with long-standing persistent AF.
▪ AF duration and PV isolation along with potential AF driver ablation both were independent predictors of recurrent AF after catheter ablation of long-standing persistent AF.

## Introduction

Atrial fibrillation (AF) has been the most frequently occurring, sustained arrhythmia in the older adults and causes significant morbidity and mortality.^1^ AF can be maintained by stable and rapid reentrant circuits resulting in fibrillatory conduction throughout the atria.^2^, ^3^, ^4^ Catheter ablation that targets regions with fractionated potentials or high frequencies during AF has been proposed as a treatment strategy.^4^, ^5^, ^6^, ^7^ However, the benefit of adjuvant complex fractionated atrial electrograms (EGMs) or linear ablation after successful circumferential pulmonary vein isolation (PVI) is controversial based on recent data from STAR AF II (Substrate and Trigger Ablation for Reduction of Atrial Fibrillation Trial Part II).^8^ In addition, adjuvant elimination of drivers and non-PV triggers has been proposed as a potential strategy in patients with persistent and long-standing persistent AF.^9^ Lin et al^10^ demonstrated that in patients with persistent and long-standing persistent AF, a specified substrate modification guided by targeting the presumed AF drivers could result in a higher AF termination rate, fewer substrate ablation lesions, and lower recurrent AF. A meta-analysis showed that AF driver-guided catheter ablation could increase freedom from AF relative to conventional strategies in patients with nonparoxysmal AF.^11^ Therefore, the optimal ablation strategy for long-standing persistent AF remains undetermined.

AF propagation maps based on direct contact mapping (without phase transformation) using dedicated software (CARTOFINDER; Biosense Webster Inc., Diamond Bar, CA) also have been reported.^12^^,^^13^ High-resolution, sequential endocardial EGM-based mapping could allow identification of focal and rotational activities in persistent AF. Honarbakhsh et al^14^ showed that the CARTOFINDER system could effectively identify focal activities during mapping of persistent AF, and ablation of these focal activities would be a vital therapeutic target in ablation of persistent AF.

However, the long-term outcome of ablation of these focal and rotational activities in patients with long-standing persistent AF has not been evaluated systematically. Therefore, in the present study, we aimed to investigate the long-term outcomes of patients with long-standing persistent AF using the CARTOFINDER system.

## Methods

### Study population

**Figure 1:**
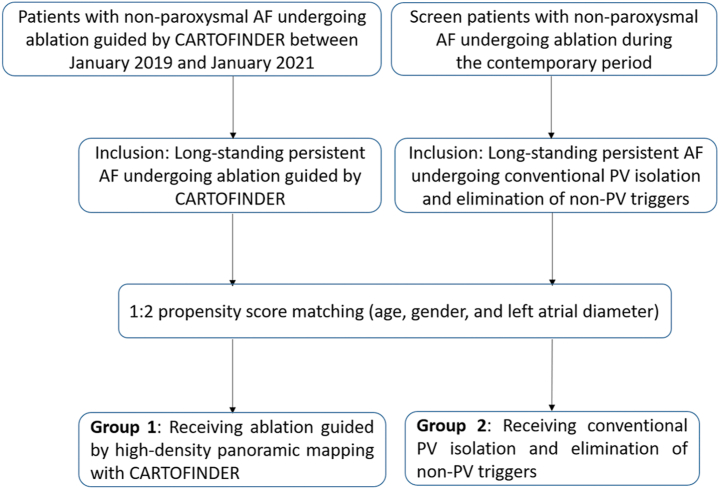
Enrollment of the study population. AF = atrial fibrillation; PV = pulmonary vein.

Between January 2019 and January 2021, a total of 76 patients with symptomatic and drug-refractory nonparoxysmal AF referred to Taipei Veterans General Hospital for ablation guided by a novel high-density panoramic mapping system with CARTOFINDER were prospectively enrolled. Of this cohort, 40 patients (52.6%) had long-standing persistent AF (CARTOFINDER group). We then retrospectively screened the patients with long-standing persistent AF undergoing conventional PVI and elimination of non-pulmonary vein (non-PV) triggers during the contemporary period as a conventional group. Propensity score analysis was used to adjust for 3 confounding factors, including age, sex, and left atrial (LA) diameter. They were matched at a 1:2 ratio, which resulted in 2 balanced groups (40 patients in group 1 received ablation-guided high-density panoramic mapping system with CARTOFINDER; 80 patients in group 2 received conventional PVI and elimination of non-PV triggers). Long-standing persistent AF was defined according to the 2017 Heart Rhythm Society Expert consensus document.^15^ Figure 1 shows the enrollment of the study population. All patients presented with AF at the onset of the procedure.

### Study design

This study was conducted at the Taipei Veterans General Hospital in Taipei after approval by the institutional review board of the Taipei Veterans General Hospital (IRB: 2016-08-022CC, 2019-02-018A). Written informed consent was obtained from all patients. The primary endpoint was recurrence of AF with an episode lasting >30 seconds, 3 months after the ablation. In addition to recurrence of AF, recurrences of atrial flutter and atrial tachycardia were analyzed in patients who underwent high-density panoramic mapping with CARTOFINDER (group 1) and those with conventional PVI and elimination of non-PV triggers (group 2). In our previous reports,^9^^,^^10^ 1-year recurrence of longstanding persistent AF after ablation was around 60%, and elimination of localized reentry and nonpulmonary focal sources after PVI could result in a better outcome (hazard ratio 0.26). In the settings of 1:2 ratio (CARTOFINDER group vs conventional group), 80% power, and 70% reduction of AF recurrence with the CARTOFINDER method compared with the conventional group, the sample size would be 36 for the CARTOFINDER group and 71 for the conventional group.^16^

### Electrophysiological study

Electrophysiological study and catheter ablation in the fasting state were performed in each patient after obtaining informed consent. All antiarrhythmic drugs, except for amiodarone, were discontinued for at least 5 half-lives before initiation of the procedure. Amiodarone was held 2 weeks before the procedure. None of the patients received amiodarone during the electrophysiological procedure. Electroanatomic mapping was performed in all patients, and a stepwise procedure for catheter ablation was used, as described in detail previously.^7^^,^^17^

### Definition of drivers in this study (including focal activity and rotational activity)

**Figure 2:**
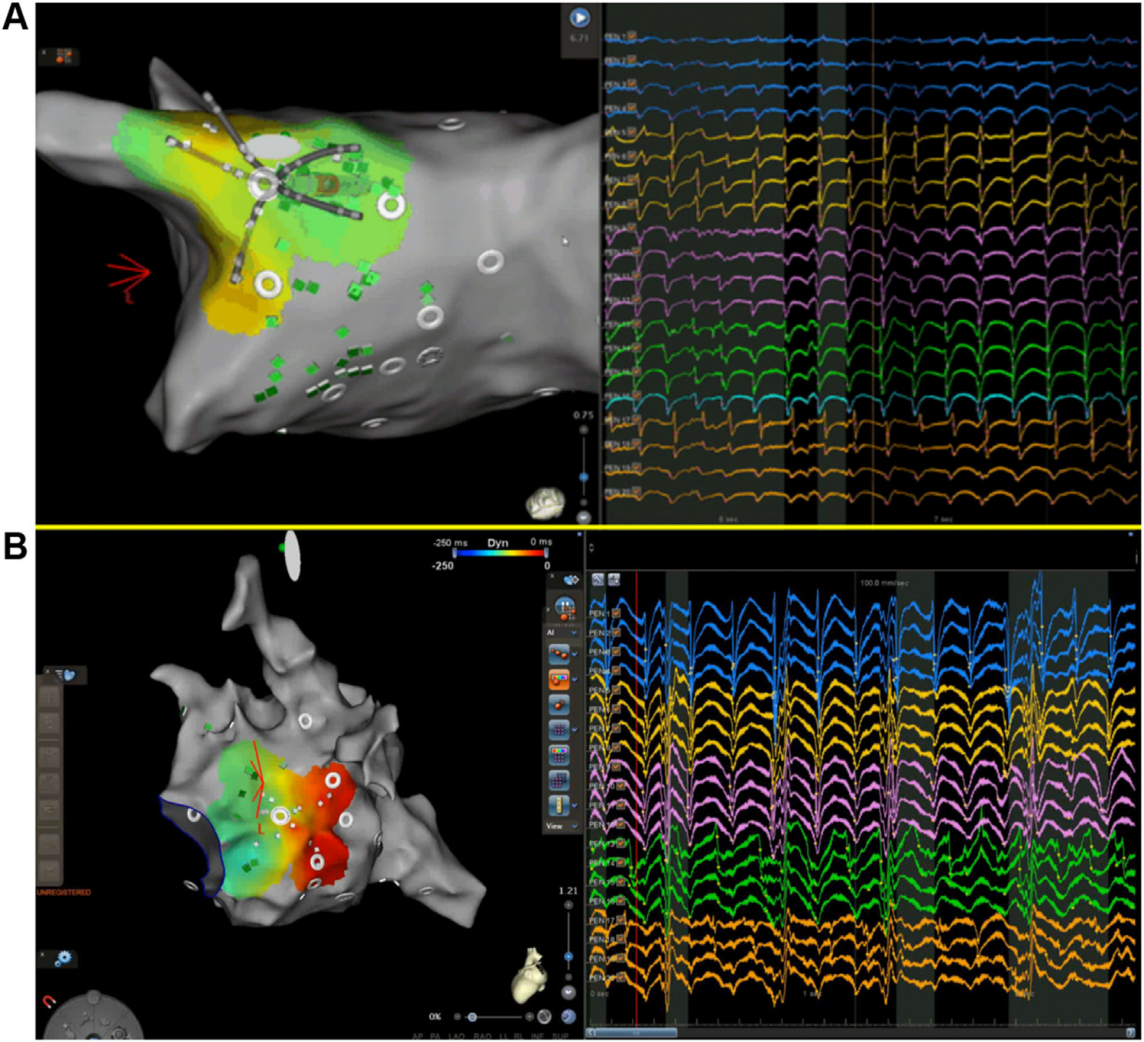
Examples of CARTOFINDER-identified activities. **A:** Focal activity near the posterior antrum of the left superior pulmonary vein. **B:** Rotational activity near the posterior mitral isthmus.

A region of interest algorithm was developed to assist operators in identifying both focal and rotational activities during mapping AF. In brief, focal activities are detected by identifying a QS wave pattern in the unipolar EGMs. If >2 early QS wave patterns are detected within a 2-second window, a focal source is considered to exist (Figure 2A).^18^

Rotational activities are detected by identifying pansystolic activations occurring within consecutive electrodes. A pansystolic activation “wave” is defined as a series of EGMs in consecutive electrodes occupying >50% of the local cycle length with a distance <20 mm between the starting and ending points of the wave. Two or more such pansystolic activations for which at least 80% of the electrodes follow the same propagation pattern are defined as rotational activity (Figure 2B).^18^

### Group 1: CARTOFINDER method

#### Step 1: Fast anatomic mapping and CARTOFINDER mapping

**Figure 3:**
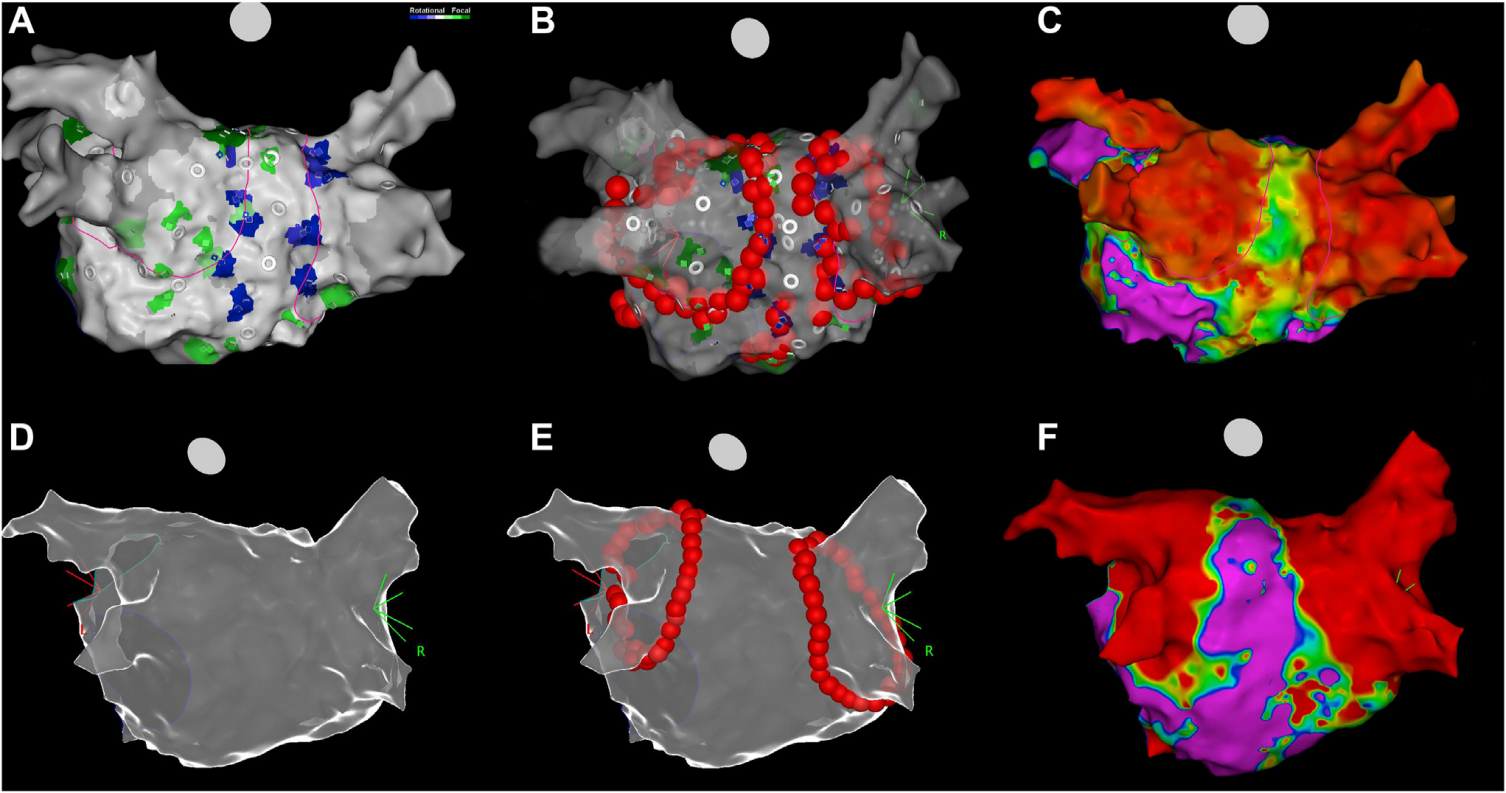
Comparison between the CARTOFINDER-guided approach and the conventional method of wide antral isolation. **A:** Fast anatomic mapping and CARTOFINDER mapping. **B:** Modified ablation line encircling the lesions identified by CARTOFINDER around the pulmonary vein antra. **C:** Bipolar voltage map during sinus rhythm after ablation guided by CARTOFINDER. **D:** Creation of electroanatomic geometry. **E:** Conventional wide antral isolation during atrial fibrillation. **F:** Bipolar voltage map during sinus rhythm after ablation by conventional wide antral isolation.

After transseptal puncture under guidance of right atriography, a fast anatomic map (FAM) of the LA and the PVs was created with a multipolar mapping catheter (PentaRay, Biosense Webster). After creation of the FAM, the panoramic mapping system using the CARTOFINDER system to identify rotational/focal activities was performed with the PentaRay catheter in the LA (Figure 3A).

#### Step 2: Modified wide antral PVI with encompassing CARTOFINDER-identified activities around the PV antra

Wide antral PVI was performed by ablation with an irrigated-tip ablation catheter at the PV antrum confirmed by elimination of local signal. Radiofrequency energy from 25–40 W was applied for 20–40 seconds for each lesion, with a target temperature <40°C. We targeted the anterior LA wall with ablation index (AI) 500 and the posterior LA wall with AI 450. If CARTOFINDER-identified focal/rotational activities are located around the PVs, the isolation line is adjusted to encircle lesions around the PV antra to encompass these CARTOFINDER-identified activities (Figure 3B).

#### Step 3: AF driver ablation using a high-density panoramic mapping system with CARTOFINDER

If the AF persisted after step 2, elimination of CARTOFINDER-identified focal/rotational activities was attempted except for those inside the left atrial appendage (LAA). Ablation at focal/rotational activities sites was delivered with power of 25–40 W, aiming for the center of the focal or rotational activation. Further ablation was delivered in a cluster of lesions surrounding the first point. Ablation in any region was stopped if AF was terminated or no signal remained in the area of focal/rotational activities. The ablation time in any 1 cluster was restricted to 5 minutes. Care was taken not to form a linear lesion so as not to impact any AF mechanisms. Other than isolating PVs and targeting potential drivers, no additional ablation was performed during AF. If the AF organized into an atrial tachycardia or atrial flutter, this was mapped and ablated. If AF persisted even after these procedures, sinus rhythm was restored with electrical cardioversion (Figure 3C).

Right atrial (RA) cavotricuspid isthmus ablation was routinely performed at the end of the AF procedure. Bidirectional conduction block of linear ablation was demonstrated during sinus rhythm.

### Group 2: Conventional method

#### Step 1: Wide antral PVI

After transseptal puncture under the guidance of right atriography, a decapolar circular or multielectrode catheter was placed in the LA through femoral venous access. The electroanatomic geometry of the LA was constructed using a 3-dimensional navigation system (NavX, Abbott Medical, Minnetonka, MN; or CARTO3, Biosense Webster, Diamond Bar, CA) (Figure 3D). Continuous circumferential lesions were created by encircling the atrial side of the bilateral PV antra with an irrigated-tip ablation catheter (Figure 3E). Radiofrequency energy 25–40 W was applied for 20–40 seconds for each lesion, with a target temperature <40°C. With the CARTO3 System, we targeted the anterior LA wall with AI 500 and the posterior LA wall with AI 450. If the AF persisted after PVI , sinus rhythm was restored using electric cardioversion (Figure 3F).

#### Step 2: Non-PV trigger ablation

After restoration of sinus rhythm, spontaneous onsets of atrial ectopic beats were recorded, or repeat short runs of sustained AF would be facilitated to initiate AF with or without isoproterenol infusion (4–8 μg/min). Intermittent overdrive pacing from the RA, coronary sinus (CS), and PV or administration of intravenous high-dose adenosine (18–24 mg) was performed to induce atrial ectopy in patients without initial atrial ectopy or ectopy that was noninducible with isoproterenol infusion (4–8 μg/min). Continued overdrive pacing was performed until sustained AF was induced.

The location of non-PV ectopy was evaluated using the activation sequence of the high RA, His-bundle area, and CS. Measuring the time difference between high RA and His-bundle area activation during sinus rhythm and ectopy (ectopic beat <0 ms) can help reveal the site of ectopy, differentiating between the superior vena cava, upper crista terminalis, and PVs. For mapping of non-PV triggers from the LA, if the earliest activation site was near the left PV ostium or posterolateral portion of the mitral annulus, differential pacing was performed to differentiate the ectopic beats from the vein of Marshall. Catheter ablation was conducted using a 4-mm-tip ablation catheter or an irrigated-tip ablation catheter with a target temperature <40°C (duration 40 seconds for each lesion) toward the earliest electrical activity or a local unipolar QS pattern of the ectopic beats preceding the onset of AF. The endpoint of non-PV trigger ablation was disconnection between the superior vena cava and RA as well as between the CS and RA, and elimination of other non-PV ectopic beats with negative provocation of AF.

RA cavotricuspid isthmus ablation was routinely performed at the end of the AF procedure. Bidirectional conduction block of linear ablation was demonstrated during sinus rhythm.

### Follow-up of AF recurrence

After discharge, patients underwent follow-up 2 weeks after the catheter ablation then every 1–3 months thereafter at our cardiology clinic or with the referring physician. During each follow-up, 24-hour Holter monitoring or cardiac event recording was performed for 1 week. Recurrence was defined as recurrence of AF, atrial flutter, and atrial tachycardia, and was defined as an episode lasting >30 seconds, 3 months after the ablation.

### Statistical analysis

Patient characteristics are given as mean ± SD for continuous variables and as frequency (percentage) for categorical variables. Continuous and categorical variables were compared using the Student *t* test and the χ^2^ test with Yates correction, respectively. Proportions were compared using the χ^2^ test or Fisher exact test, as appropriate. One-way analysis of variance was used to compare data among the 2 groups. Propensity score matching was used to control for confounders.^19^^,^^20^ The propensity score was obtained using logistic regression. The recurrence-free survival curve was examined using the Kaplan-Meier method with log-rank test. Cox proportional hazards regression was also used to compare the risk among the 2 groups, with results expressed as hazard ratio with 95% confidence interval. Statistical significance was set at *P* <.05. Statistical analyses were performed using IBM SPSS 20 for Windows (SPSS, Inc., Chicago, IL).

## Results

### Baseline characteristics and mapping details between the two propensity score matching groups with long-standing persistent AF

Baseline characteristics and demographics of the 2 groups are listed in Table 1. Age, sex, body mass index, LA diameter, CHA_2_DS_2_-VASc score, and AF duration were similar between the 2 groups.

**Table 1:** Baseline characteristics and demographics between the two groups with long-standing persistent AF

	Group 1 (n = 40)	Group 2 (n = 80)	*P* value
Age (y)	57.5 ± 9.3	56.5 ± 8.9	.590
BMI (kg/m^2^)	25.2 ± 6.5	25.9 ± 2.5	.489
Male	34 (85.0)	73 (91.3)	.355
Diabetes mellitus	5 (12.5)	7 (8.8)	.531
CHF	4 (10.0)	10 (12.5)	.772
Hypertension	15 (37.5)	38 (47.5)	.334
CAD	3 (7.5)	7 (8.8)	1
Ischemic stroke	3 (7.5)	12 (15.0)	.380
CHA₂DS₂-VASc score	1.26 ± 1.2	1.06 ± 1.0	.426
AF duration (mo)	39.7 ± 27	40.9 ± 26	.758
LDL (mg/dL)	107 ± 30	106 ± 18	.898
LA diameter (mm)	47.2 ± 5.1	45.9 ± 4.3	.221
Procedural time (min)	219 ± 70	150 ± 49	<.001
Patients with PV CARTOFINDER-identified activity	29 (72.5)	—	—
Termination of AF during PV isolation	1 (2.5)	3 (3.8)	.719
Patients with non-PV trigger or CARTOFINDER-identified activity	38 (95.0)	15 (18.8)	<.001
Termination of AF during non-PV atrial ablation	2 (5.0)	—	—
Acute reconnection of PV	2 (5.0)	4 (5.0)	1
Additional ablation area of CARTOFINDER-identified activity around RPV antrum (cm^2^)	0.53 ± 0.8	—	—
Additional ablation area of CARTOFINDER-identified activity around LPV antrum (cm^2^)	0.79 ± 1.4	—	—
Complications	1 (2.5)	2 (2.5)	1
Tamponade	0 (0)	0 (0)	—
Pseudoaneurysm	1 (2.5)	2 (2.5)	—
Phrenic nerve injury	0 (0)	0 (0)	—
Amiodarone, before ablation	22 (55.0)	46 (51.7)	—
Amiodarone, after ablation	14 (35.0)	36 (45.0)	—
No. of AADs during follow-up	1.1 ± 0.8	1.2 ± 0.6	.517
Recurrence of AF	12 (30.0)	56 (70.0)	<.001
Recurrence of atrial flutter	6 (15.0)	20 (25.0)	.247
Recurrence of atrial tachycardia	0 (0)	2 (2.5)	.552

Values are given as mean ± SD or n (%) unless otherwise indicated.

AAD = antiarrhythmic drug; AF = atrial fibrillation; BMI = body mass index; CAD = coronary artery disease; CHF = congestive heart failure; LA = left atrium; LDL = low-density lipoprotein; LPV = left pulmonary vein; PV = pulmonary vein; RPV = right pulmonary vein.

In group 1, a mean of 6.7 ± 4.1 focal (total PV: 73; non-PV: 195) and 0.4 ± 0.9 rotational activities (total PV: 10; non-PV: 5) were observed per patient. Twenty-nine patients (72.5%) had PV focal or rotational activities, 38 patients (95.0%) had non-PV focal or rotational activities, and 32 (80.0%) patients had only focal activities.

In group 2, a mean of 0.2 ± 0.5 non-PV triggers (total non-PV: 19) were identified after restoration of sinus rhythm with PVI or cardioversion. Fifteen patients (18.8%) had non-PV triggers, including 7 (8.8%) in the superior vena cava, 2 (2.5%) in the CS ostium, 3 (3.8%) in the vein of Marshall, and 7 (8.8%) in the LA septum.

**Figure 4:**
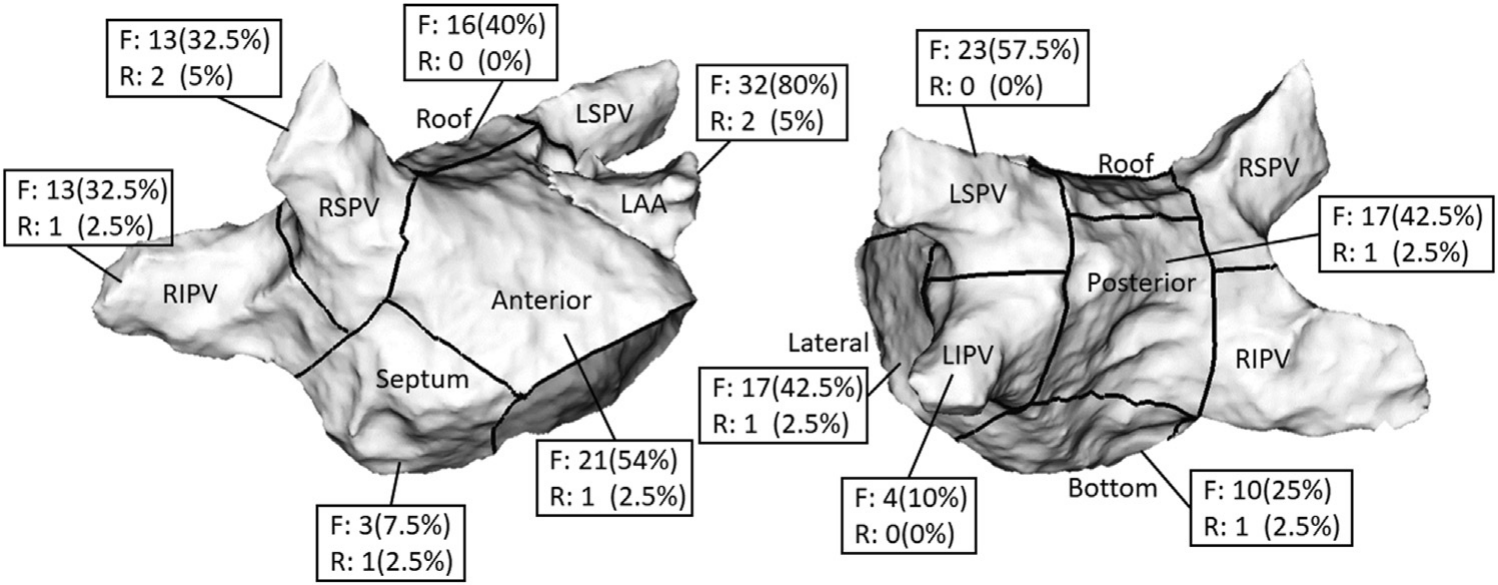
Distribution of focal (F) and rotational (R) activities of long-standing persistent atrial fibrillation in group 1. The left atrium was divided into 11 regions: right superior pulmonary vein (RSPV), right inferior pulmonary vein (RIPV), left superior pulmonary vein (LSPV), left inferior pulmonary vein (LIPV), left atrial appendage (LAA), septum, anterior wall, roof, posterior wall, bottom, and lateral wall. The number and percentage of patients in group 1 with CARTOFINDER-identified focal/rotational activities in each region were calculated. For example, in group 1, 32 patients (80%) had focal activities and 2 patients (5%) had rotational activities in the LAA.

The distribution of different regions of LA with CARTOFINDER-identified focal/rotational activities in group 1 is shown in Figure 4. The number and percentage of patients in group 1 with focal/rotational activities in each region were calculated. The most common areas detected with focal activities were the LAA base (32 patients [80%]), left superior PV (23 patients [57.5%]), and anterior wall of the LA (21 patients [54%]), whereas the most common areas detected with rotational activities were the LAA base (2 patients [5%]) and right superior PV (2 patients [5%]).

#### Procedural details and response to radiofrequency catheter ablation

During catheter ablation of AF, PVI was achieved in all patients of both groups. Procedural time was longer in group 1 than in group 2 (219 ± 70 minutes vs 150 ± 49 minutes, respectively; *P* <.001).

During PVI, procedural termination of AF to sinus rhythm could be observed in 1 patient (2.5%) of group 1 and 3 patients (3.8%) of group 2 (*P* = .719). During non-PV atrial ablation, acute termination of AF could be observed in 2 patients (5.0%) of group 1. Overall, the rate of AF termination during the whole procedure was similar between the 2 groups (3 [7.5%] in group 1 and 3 [3.8%] in group 2; *P* = .374). The rate of acute reconnection of PV during the procedure also was similar between the 2 groups (2 [5.0%] in group 1 and 4 [5.0%] in group 2; *P* = 1).

During the stepwise approach of the CARTOFINDER method, an average of 28.6% (81/283) of CARTOFINDER-identified activities was located near the PV antra and could be eliminated by a modified line of wider antral PVI. Meanwhile, 21.6% (61/283) of CARTOFINDER-identified activities were not ablated because of their location within the LAA, and those 4.6% (13/283) of activities also were not ablated because of AF termination. The 45.2% (128/283) of remaining activities were eliminated at step 3 AF driver ablation using panoramic mapping system with CARTOFINDER.

### Safety characteristics between the two groups

The overall major complication rate was low and comparable between the 2 groups (1 [2.5%] pseudoaneurysm in group 1 and 2 [2.5%] pseudoaneurysms in group 2). The safety characteristics of the 2 groups are given in Table 1.

### Long-term outcome during clinical follow-up

**Figure 5:**
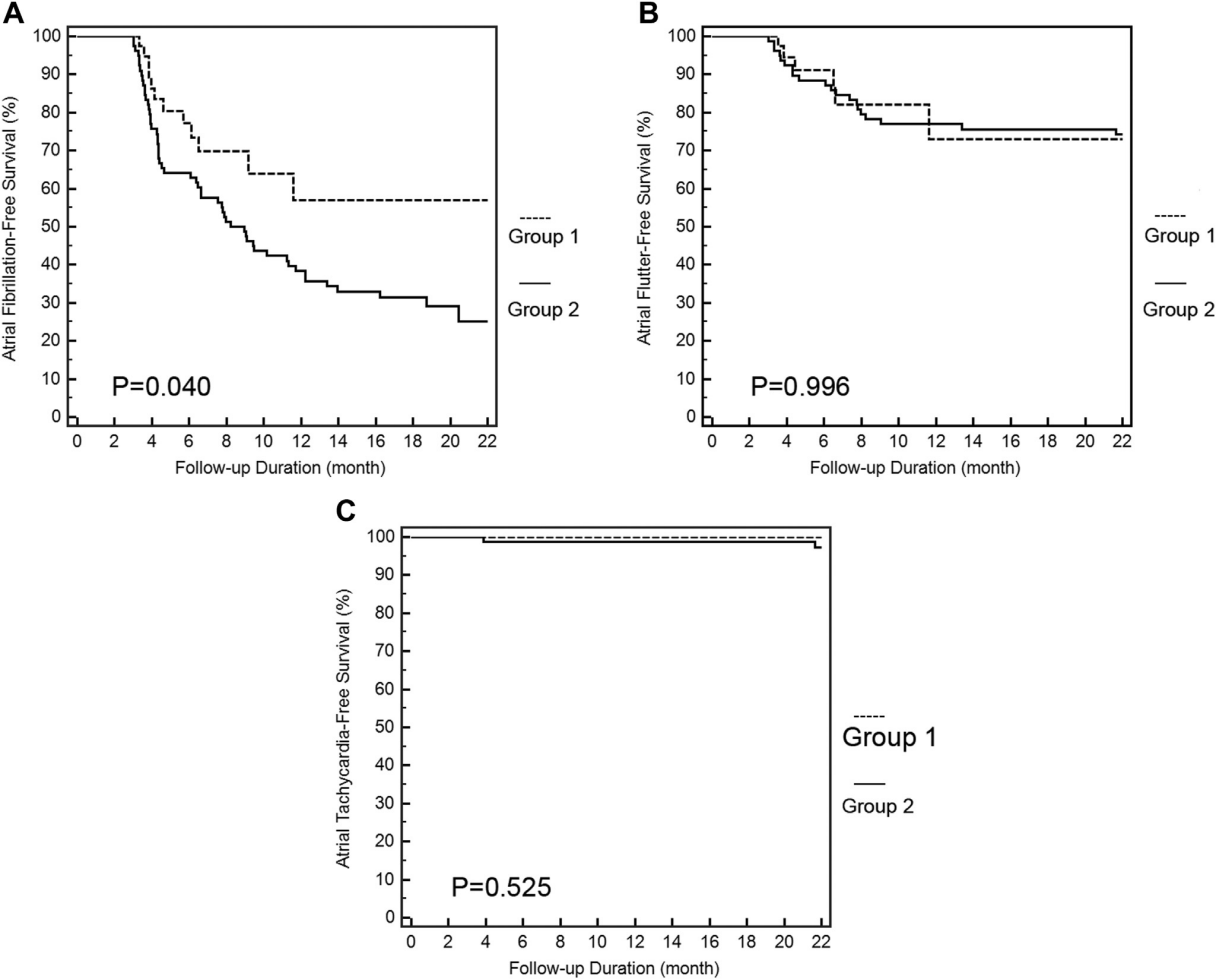
Kaplan-Meier curves of recurrence after the index procedure during follow-up between the 2 groups. **A:** Recurrence of atrial fibrillation between group 1 (CARTOFINDER method) and group 2 (conventional method). **B:** Recurrence of atrial flutter between group 1 (CARTOFINDER method) and group 2 (conventional method). **C:** Recurrence of atrial tachycardia between group 1 (CARTOFINDER method) and group 2 (conventional method).

With mean follow-up of 12.2 ± 7.9 months, patients in group 1 had a lower AF recurrence rate than those in group 2 (12 [30%] in group 1 vs 56 [70%] patients in group 2; log-rank *P* = .040) (Figure 5A). The mean number of antiarrhythmic drugs was similar between the 2 groups (1.1 ± 0.8 in group 1 and 1.2 ± 0.6 in group 2; *P* = .517). There also was no difference in recurrence of atrial flutter (6 [15%] patients in group 1 and 20 [25%] patients in group 2; log-rank *P* = .996) (Figure 5B) and atrial tachycardia (0 [0%] patient in group 1 and 2 [2.5%] patients in group 2; log-rank *P* = .525) (Figure 5C).

### Predictors of long-term outcome

Compared to patients without recurrent AF, those with recurrent AF had longer AF duration, and 82.1% of patients underwent the procedure with a conventional method (Table 2). In Cox proportional hazards regression analysis, AF duration and PVI along with AF driver ablation using a panoramic mapping system with CARTOFINDER both were independent predictors of recurrent AF after catheter ablation of long-standing persistent AF (Table 2).

**Table 2:** Comparison of factors between patients with/without recurrent AF and Cox proportional hazards regression analysis of AF recurrence after catheter ablation

	Without recurrent AF	With recurrent AF	*P* value	Univariate analysis		Multivariate analysis	
	(n = 52)	(n = 68)		*P* value	95% CI	*P* value	95% CI
Age (y)	57.2 ± 10.9	56.7 ± 7.5	.768	.975	0.976–1.024	—	—
BMI (kg/m^2^)	25.5 ± 5.8	23.8 ± 8.1	.456	.941	0.874–1.013	—	—
Male	45 (86.5)	62 (91.2)	.555	.648	0.525–2.816	—	—
Diabetes mellitus	4 (7.7)	8 (11.8)	.550	.260	0.729–3.219	—	—
CHF	7 (13.5)	7 (10.3)	.775	.360	0.316–1.518	—	—
Hypertension	17 (32.7)	36 (52.9)	.041	.161	0.873–2.269	—	—
CAD	4 (7.7)	7 (10.3)	.755	.184	0.777–3.732	—	—
Ischemic stroke	7 (13.5)	8 (11.8)	.788	.536	0.378–1.658	—	—
CHA₂DS₂-VASc score	1.17 ± 1.3	1.10 ± 1.0	.747	.678	0.777–1.178	—	—
AF duration (mo)	30.3 ± 30	45.9 ± 40	.021	.020	1.001–1.013	.022	1.001–1.013
LDL (mg/dL)	106.9 ± 26	107.6 ± 21	.936	.661	0.969–1.051	—	—
LA diameter (mm)	46.1 ± 4.1	46.3 ± 4.9	.779	.514	0.963–1.078	—	—
Group 1	28 (53.8)	12 (17.6)	<.001	.044	0.280–0.982	.046	0.282–0.989
Termination of AF during ablation	3 (5.8)	3 (4.4)	1	.876	0.343–3.504	—	—
Acute reconnection of PV	3 (5.8)	3 (4.4)	1	.876	0.343–3.504	—	—
Procedural time (min)	193.9 ± 71	153.8 ± 54	.005	.223	0.979–1.005	—	—
No. of AADs	1.1 ± 0.7	1.2 ± 0.5	.524	.921	0.695–1.496	—	—

Values are given as mean ± SD or n (%) unless otherwise indicated.

CI = confidence interval; other abbreviations as in Table 1.

## Discussion

The main findings of this study were as follows. (1) High-density panoramic mapping with CARTOFINDER can identify focal and rotational activities and assist in modifying wide-area PV ablation by encompassing CARTOFINDER-identified activities around the PV antra. (2) Compared with conventional PVI and elimination of non-PV triggers, the stepwise approach of the CARTOFINDER method resulted in less recurrent AF in patients with long-standing persistent AF. (3) AF duration and PVI along with AF driver ablation using a panoramic mapping system with CARTOFINDER both were independent predictors of recurrent AF after catheter ablation of long-standing persistent AF.

### Comparison with results of previous studies: Distribution of focal/rotational activities, procedural termination of AF, and long-term outcomes

In a study of 49 patients with nonparoxysmal AF (38% with long-standing persistent AF) undergoing ablation guided by the CARTOFINDER system, Takahashi et al^21^ showed that LA focal/rotational activities were mostly identified in the LAA and the inferior and lateral LA. Our results also revealed that the most common area detected with focal/rotational activities was the LAA base (Figure 4).

In patients with persistent AF, Honarbakhsh et al^14^ described AF termination in 12 of 19 patients (63%) after CARTOFINDER-guided ablation of AF drivers. In more advanced remodeling stages, acute procedural success in terminating AF was low in the study of Calvo et al,^22^ who used the CARTOFINDER system in patients with long-standing persistent AF. Our acute procedural success in terminating AF was similar to that reported by Calvo et al,^22^ and procedural termination of AF was not related to long-term recurrent AF in our study cohort (Table 2). Although the rate of AF termination may be viewed as an endpoint of acute procedural success, recent data have questioned its role in predicting recurrence of AF during long-term follow-up and therefore should be reconsidered with caution.^18^

Verma et al^8^ demonstrated equivalent efficacy of circumferential PVI compared to additional linear ablation or complex fractionated atrial electrogram–guided ablation in STAR AF II. Despite these clinical results, in patients with long-standing persistent AF, it was related to a high recurrence rate (60% within 1 year) due to atrial dilation and extensive substrate remodeling. Nevertheless, in studies of nonparoxysmal AF ablation guided by the CARTOFINDER system, reported recurrence rates ranged from 18%–47%.^14^^,^^18^^,^^20^ Our results showed that recurrence of AF was 30.8% in patients undergoing a procedure guided by the CARTOFINDER system and 70.5% in those undergoing a procedure with a conventional method. This difference might result from the more effective identification and ablation of drivers of AF. However, the improvement in outcome was limited to reduction of AF recurrence and not of atrial flutter or atrial tachycardia recurrence. This could be due to residual AF drivers that either were not eliminated because of restoration of sinus rhythm during ablation or were located deep inside the LAA.

### Potential therapeutic target of patients with long-standing persistent AF beyond PVI

Takahashi et al^23^ demonstrated that CARTOFINDER-identified focal activities were observed most frequently in the LAA. In our study, 32 patients (80%) in group 1 had focal activities and 2 patients (5%) had rotational activities in the LAA. The LAA has been shown to play a vital role in the initiation and maintenance of atrial arrhythmias.^24^ Furthermore, a study reported that PVI and substrate modification did not affect focal or rotational activities inside the LAA.^25^ This result might suggest focal/rotational activities in the LAA to be one of the dominant mechanisms for the maintenance of AF beyond the PVs. Because of the risk of periprocedural complications, we did not routinely ablate activities inside the LAA. LAA isolation/exclusion might have benefits on clinical outcomes in selected patients with persistent AF,^26^^,^^27^ but the side effects and complications of these interventions should be taken into consideration.^27^, ^28^, ^29^ For the reasons of efficacy and safety, further studies are warranted to determine whether LAA isolation or exclusion in patients with identified focal/rotational activities inside the LAA is safe and results in improvement in terms of freedom from recurrent atrial arrhythmias.

### Focused substrate modification by elimination of potential drivers of long-standing persistent AF

Notwithstanding the fact that circumferential PVI alone might not be enough for patients with long-standing persistent AF, adding more extra-PV atrial ablation could result in more inevitable scar formation^30^ and increase the risk of complications. Gibson et al^31^ reported that pulmonary hypertension after catheter ablation could be found in 1.4% of patients without PV stenosis and it was associated with elevated LA pressure, large LA scar area, diabetes, and obstructive sleep apnea. The DECAAF (Delayed Enhancement-magnetic resonance imaging determinant of successful Catheter Ablation of Atrial Fibrillation) study also showed higher recurrent AF after catheter ablation in patients with extensive atrial scar.^32^ Park et al^33^ demonstrated that electroanatomic remodeling of the LA was an independent predictor for recurrence after AF ablation. Therefore, balanced substrate modification with limited tissue damage is crucial to achieving a better long-term clinical outcome and minimizing procedure-related complications.

Although there were no significant difference in recurrence of atrial flutter and atrial tachycardia, our study indicated that patients in group 1 had lower recurrent AF than patients in group 2. With the new algorithm, efficient identification of focal and rotational drivers of AF could be achieved and be ablated effectively. Limited and focused substrate modification guided by the CARTOFINDER system could decrease tissue damage and result in less subsequent LA scar formation and possible procedure-related complications.

### Clinical perspectives for ablation of long-standing persistent AF

Ablation of persistent AF with linear ablation or substate ablation using defractionation has yielded modest clinical results; sometimes it could result in extensive ablation and potentially ensuing detrimental effects, such as deteriorated atrial hemodynamic function and proarrhythmic consequences.^34^ These unmet needs encourage the development of new ablation strategies with a focus on targeting patient-specific AF drivers. To the best of our knowledge, this is the first study to compare the long-term outcome of ablation in patients with long-standing persistent AF in propensity score matching groups. Direct endocardial high-density contact mapping EGM analysis during ongoing AF could allow the identification of more potential drivers of AF. High-density panoramic mapping with CARTOFINDER can also assist in modifying wide-area PVI by encompassing focal/rotational activities around the PV antra. Compared with conventional PVI and elimination of non-PV triggers, the stepwise CARTOFINDER method results in less recurrent AF in patients with long-standing persistent AF. A randomized study on the potential benefit of driver ablation guided by a high-density panoramic mapping system would be warranted.

### Study limitations

First, the integrated CARTOFINDER module is a novel software used for catheter ablation in persistent AF. The clinical efficacy and safety are still under investigation. In the present study, we discussed the efficacy and safety with patients in the clinics. Patients who agreed to join this study and were willing to undergo at least 12 months of follow-up were enrolled. Therefore, this study had nonconsecutive enrollment. We initiated this clinical study by using the novel CARTOFINDER module to test the efficacy in catheter ablation of patients with persistent AF. Further prospective randomized studies might be warrant after establishing the efficacy and safety in this present study. Second, although we used the reverse U curve to locate the Penta-Ray in the septum and the bottom, which would improve stability in these areas, over/underestimation of focal/rotational activities still was possible. Third, because of prolonged procedural time with CARTOFINDER mapping, RA mapping was not routinely performed. Arrhythmogenicity in the RA might have been underestimated. Fourth, the PentaRay catheter seems not to be sufficient in mapping rotational activation and may underestimate the incidence and prevalence of the rotor. Fifth, evaluation of asymptomatic episodes of AF was difficult, even when Holter or cardiac event recorders were used. Della Bella et al^35^ reported that asymptomatic recurrence accounted for 17% of early recurrences. Sixth, some patients were followed with a 24-hour Holter monitor but others were followed with 1-week recordings of a cardiac event recorder. The difference in ECG monitoring during follow-up and the duration of follow-up between the 2 groups might impact the recurrence.

## Conclusion

Identification of potential non-PV drivers in long-standing persistent AF is crucial, and CARTOFINDER can assist in modifying the wide-area isolation around the PV antra. Compared with the conventional method, application of CARTOFINDER might have better outcomes in patients with long-standing persistent AF.
